# Construction of miRNA-target networks using microRNA profiles of CVB3-infected HeLa cells

**DOI:** 10.1038/s41598-019-54188-w

**Published:** 2019-11-29

**Authors:** Hai Lan Yao, Mi Liu, Wen Jun Wang, Xin Ling Wang, Juan Song, Qin Qin Song, Jun Han

**Affiliations:** 1 Department of Biochemistry & Immunology, Capital Institute of Pediatrics, 2 YaBao Rd, Beijing, 100020 China; 2 State Key Laboratory of Infectious Disease Prevention and Control, Collaborative Innovation Center for Diagnosis and Treatment of Infectious Diseases, National Institute for Viral Disease Control and Prevention, Chinese Center for Disease Control and Prevention, 155 Changbai Rd, Beijing, 102206 China; 3 Center for Biosafety Mega-Science, Chinese Academy of Science, 44 Xiao HongShan, Wuhan, Hubei 430071 China

**Keywords:** Virology, Viral host response

## Abstract

MicroRNAs (miRNAs) play an important role in regulating gene expression in multiple biological processes and diseases. Thus, to understand changes in miRNA during CVB3 infection, specific miRNA expression profiles were investigated at 3 h, 6 h, and 9 h postinfection in HeLa cells by small-RNA high-throughput sequencing. Biological implications of 68 differentially expressed miRNAs were analyzed through GO and KEGG pathways. Interaction networks between 34 known highly differentially expressed miRNAs and targets were constructed by mirDIP and Navigator. The predicted targets showed that *FAM135A*, *IKZF2*, *PLAG1*, *ZNF148*, *PHC3*, *LCOR* and *DYRK1A*, which are associated with cellular differentiation and transcriptional regulation, were recognized by 8 miRNAs or 9 miRNAs through interactional regulatory networks. Seven target genes were confirmed by RT-qPCR. The results showed that the expression of *DYRK1A*, *FAM135A*, *PLAG1*, *ZNF148*, and *PHC3* were obviously inhibited at 3 h, 6 h, and 9 h postinfection. The expression of *LCOR* did not show a significant change, and the expression of *IKZF2* increased gradually with prolonged infection time. Our findings improve the understanding of the pathogenic mechanism of CVB3 infection on cellular differentiation and development through miRNA regulation, which has implications for interventional approaches to CVB3-infection therapy. Our results also provide a new method for screening target genes of microRNA regulation in virus-infected cells.

## Introduction

Enteroviruses include diverse RNA viruses classified in the *Picornaviridae* family. The genus Enterovirus consists of 15 species, including enteroviruses, polioviruses, coxsackieviruses, rhinoviruses, and echoviruses^1^. Different species of enterovirus can cause multiple human diseases, such as Poliovirus (PV)-associated infantile paralysis, coxsackievirus B3 (CVB3)-associated myocarditis, enterovirus 71 (EV71) and other enterovirus-associated hand foot and mouth disease, enterovirus 68 (EV68) and rhinovirus-associated respiratory diseases^2^.

CVB3 is one of the major causative agents of viral myocarditis, which can eventually lead to heart failure and dilated cardiomyopathy, resulting in nearly 50% of heart transplantation in children and young adults^3–7^. CVB3 also infects the human brain and pancreas and contributes to aseptic meningitis and pancreatitis^3^. The main pathological processes of viral myocarditis and pancreatitis are early direct virus-induced cytopathic effects and severe inflammatory injury, followed by host immune responses. However, the exact pathogenesis of CVB3 is not fully understood.

MicroRNAs (miRNAs) are a highly conserved class noncoding RNA with a length of approximately 18–25 nucleotides^8,9^. miRNAs play a key role in regulating cellular development, proliferation, differentiation, cellular growth control and disease progression^10,11^. Host miRNAs regulate various signal pathways to mediate host–virus interactions during viral infection. Viruses induce the expression of certain cellular miRNAs to interfere with cellular function. miRNAs influence the cellular tropism of viruses, modulate viral infectivity, and induce appropriate antiviral immune responses^12–16^. The role of regulatory mechanisms of host miRNAs in CVB3–host interactions have been elucidated by several studies. CVB3 infection changes the expression of host miRNAs, which effect viral replication or host cytopathogenesis^17^. Using an *in vitro* CVB3 infection model, miR-141, which is activated by early growth response protein 1 (EGR1) as the transcription factor, switches the protein translation initiation pattern through inhibition of eIF4E to benefit viral replication^18^. Expression of miR-203 promotes CVB3 replication both *in vitro* and *in vivo* through inhibition of transcription factor zinc finger protein 148 (ZNF-148), which downregulates genes of cell cycle arrest and upregulate genes of cell growth^19^. miR-126 promotes the formation and release of viral particles and contributes to viral cytopathogenicity by three specific targets: low-density lipoprotein receptor-related protein 6 (LRP6), sprouty-related, EVH1 domain-containing protein 1 (SPRED1), and Wnt1 responsive Cdc42 homology 1 (WRCH1) of ERK1/2 and Wnt/β-catenin signal pathways^20^. Additionally, miR-342–5p targets the nonstructural protein 2C region to repress viral replication, while miR-10a* directly binds the CVB3 genome 3D region to benefit CVB3 replication^21,22^.

Therefore, CVB3 can construct its own survival strategy through regulation of the expression of many specific miRNAs in its own ever-evolving process. The aim of this study was to establish miRNA-regulated networks to identify target genes in HeLa cells infected with CVB3 by small-RNA high-throughput sequencing. Our study also provides new experimental procedures to explore miRNA-regulated target genes by small-RNA high-throughput sequencing.

## Results

### MicroRNA profile of CVB3 virus-infected cells

A total of 3 × 10^6^ HeLa cells, a human malignant cervical tumor cell line, were inoculated with CVB3 (MOI = 1), cellular miRNA profiles were generated by small-RNA deep-sequencing. A total of 23,267,581, 21,829,256, 22,846,983 and 24,328,282 clean reads were obtained at the three time points and the control HeLa cells using BGISEQ-500 technology, respectively. All clean reads were subjected to sRNA databases, such as miRBase, Rfam, and siRNA, resulting in 2.2 × 10^7^, 2.0 × 10^7^, 2.1 × 10^7^, and 2.2 × 10^7^ miRNA sequence tags in three CVB3 infection time points and cell control, respectively.

**Figure 1:**
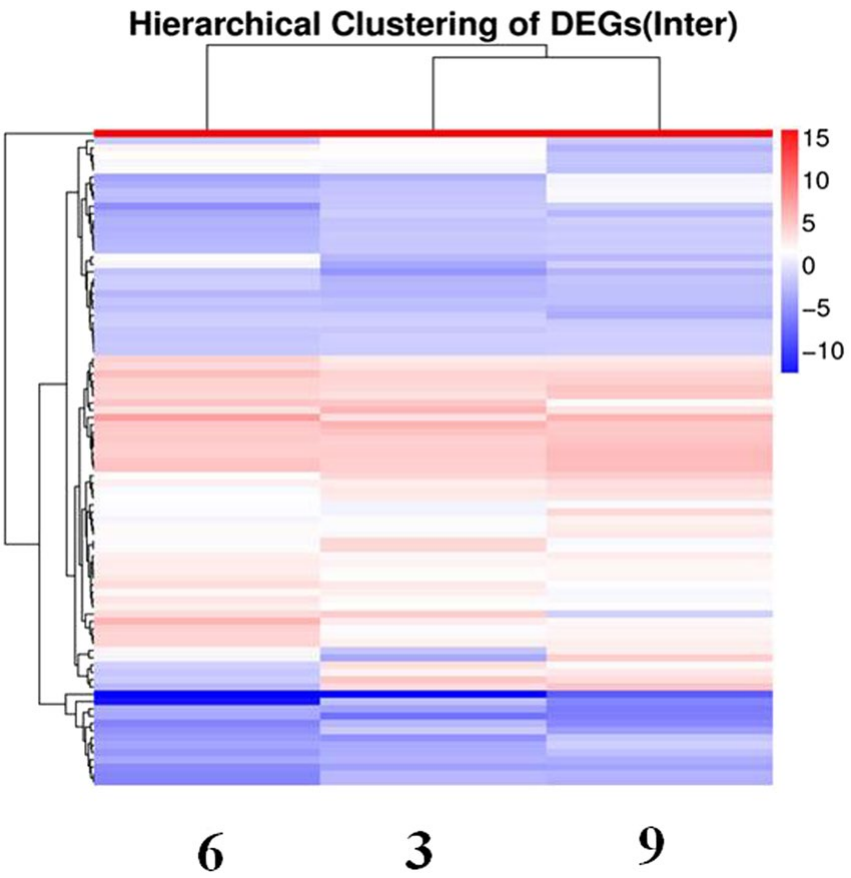
Hierarchical clustering of differentially expressed miRNAs. Hierarchical clustering was performed using differentially expressed miRNAs between CVB3-infected HeLa cells and normal cells at 3 h, 6 h and 9 h pi. The X axis represents compared miRNAs of each infection time point with normal cells. The Y axis represents DESs. Coloring indicates fold change (upregulated: red, downregulated: blue).

Compared with control cells, 970 differentially expressed miRNAs were identified in CVB3-infected cells. We used the ExpDiff method to analyze differentially expressed sRNAs (DESs) between CVB3-infected HeLa cells and normal cells at each infection time point. In this study, 597 differentially expressed miRNAs were found between CVB3-infected HeLa cells and normal HeLa cells from RNA sequencing (RNA-Seq) data. Subsequently, we further compared these 597 miRNAs among three time points and performed hierarchical clustering, as shown in Fig. 1. All differentially expressed miRNAs are shown using hierarchical clustering (Fig. 1).

### Identification of differentially expressed miRNAs in CVB3-infected HeLa cells

To reduce the range, we further compared these 597 miRNAs among three time points postinfection by >1.5 relative expression change. The expression levels of 68 miRNAs were significantly altered in CVB3-infected HeLa cells compared to uninfected HeLa cells. Of these 68 miRNAs, 51 were highly expressed, including hsa-miR-378i, hsa-miR-518e-3p, hsa-miR-422a, hsa-miR-548au-3p, hsa-miR-20b-3p, hsa-miR-150-5p, hsa-miR-4684-3p, and hsa-miR-548i, which exhibited 5-fold increased expression (Table S2). There were 17 downregulated miRNAs, including hsa-miR-522-5p, hsa-miR-32-5p, and hsa-miR-26a-1-3p (Table S2). There were 38 miRNAs that were newly identified, including novel_mir903, novel_mir898, novel_mir703, novel_mir696, novel_mir351, novel_mir343, and novel_mir334.

To verify the sequencing data, 16 miRNAs in CVB3-infected cells or normal cells were detected by RT-qPCR using TaqMan miRNA assays. RT-qPCR results were consistent with the results from sequencing data (Table S3). The high correlation between the sequencing results and RT-qPCR detection confirmed that the tested miRNAs were reproducible. The findings showed that differentially expressed miRNAs identified by small-RNA sequencing accurately reflected the miRNA content in CVB3-infected cells or normal cells.

### Analysis of Gene Ontology (GO) categories of DESs Target

To further understand the biological implications of differently expressed miRNAs, target genes of 68 miRNAs were predicted in gene term enrichment analysis using Gene Ontology (GO) categories. The analysis results showed that the predicted target genes contained the top three enriched biological processes: cellular process, regulation of biological process, single-organism process, metabolic process, and biological regulation. Within the category “cellular component”, the largest number of predicted target genes were involved in cell function, cell part, organelle and membrane function. Regarding the category “molecular function”, the predicted target genes were mainly associated with molecular transducer, catalytic activity and protein binding.

### Analysis of KEGG (Kyoto Encyclopedia of Genes and Genomes) Pathway Enrichment of DES Targets

After GO functional classification analysis, we performed pathway enrichment analysis of DES target genes based on the KEGG database and generated a report for DES target genes in each pairwise comparison. Our analysis showed that the top three were successively Signal Transduction, Global and overview maps, and Cancers. The top three parts were successively Global and overview maps, Nucleotide metabolism, Carbohydrate metabolism in Metabolism; Sorting and Degradation, Translation, Transcription in Genetic Information Processing; Cellular community, Transport and catabolism, Cell growth and death in Cellular Processes; Immune system, Endocrine system, Digestive system in Organismal Systems; Infectious Diseases and Cancers in Human Diseases; and Membrane Transport, Signal Transduction, Signaling Molecules and Interaction in Environmental Information Processing. KEGG pathway and GO category analysis demonstrated that the interaction of the target miRNAs was associated with many physiological processes.

### Prediction and confirmation of targets for multiple mirnas

**Figure 2:**
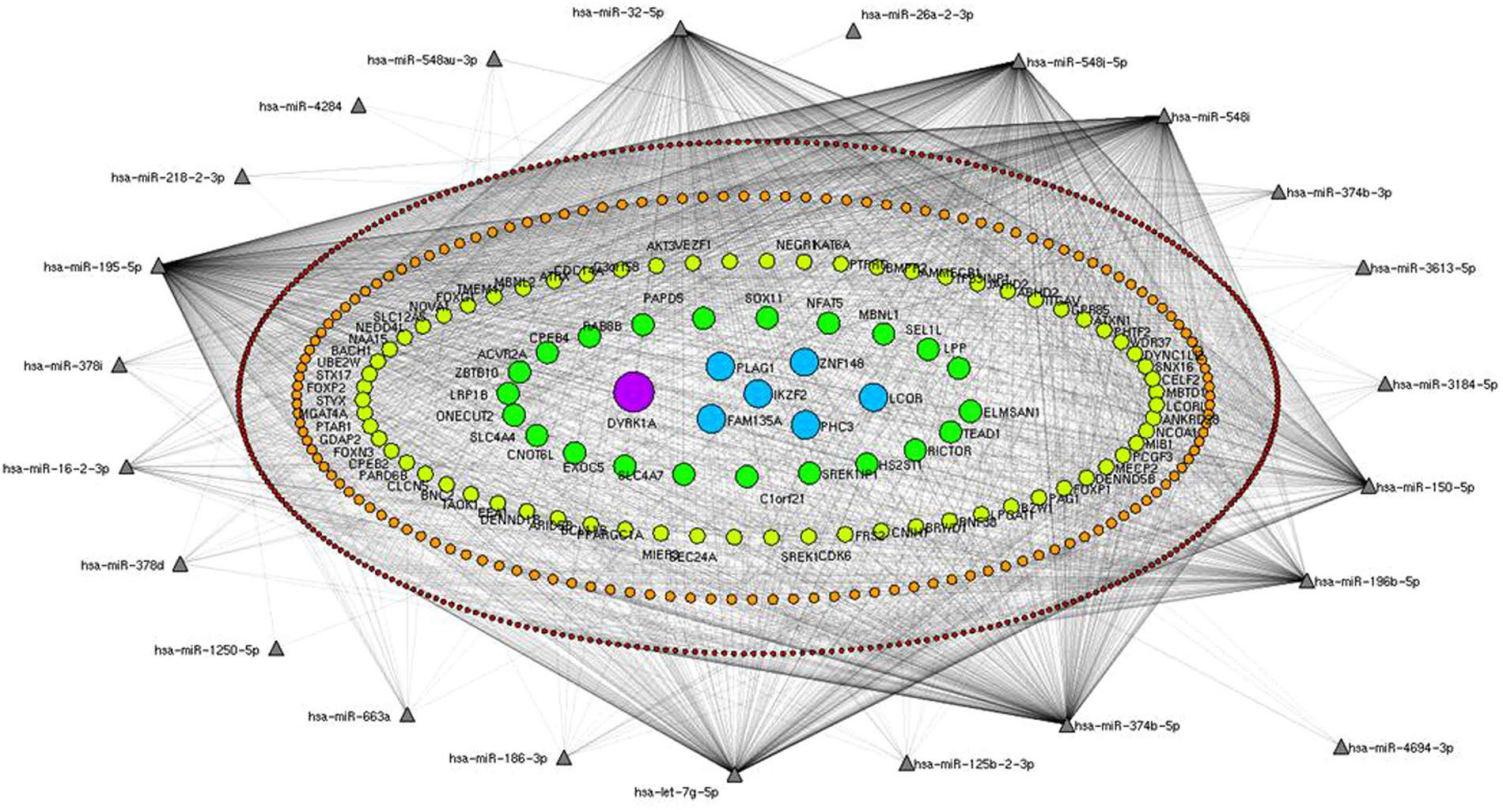
Construction of the miRNA-target network. Interaction networks between 34 known differentially expressed miRNAs and targets were established by mirDIP and Navigator. After filtration of those targets interacting with 1–3 miRNAs, targets recognized by 4 miRNAs or more were constructed into miRNA-target networks. In concentric ellipses, the targets were regulated by 4, 5, 6, 7, 8 and 9 miRNAs (deep purple circles, orange circles, light green circles, green circles, blue circles, purple circles, respectively).

Next, interaction networks between 34 known differentially expressed miRNAs (Table S4) and targets were established by mirDIP and Navigator^23,24^. We filtered and deleted those targets interacting with 1–3 miRNAs, and the target genes were identified by those regulated by 4 miRNAs or more, including hsa-miR-663a, hsa-miR-548j-5p, hsa-miR-548i, hsa-miR-548au-3p, hsa-miR-4694-3p, hsa-miR-378i, hsa-miR-378d, hsa-miR-374b-5p, hsa-miR-374b-3p, hsa-miR-32-5p, hsa-miR-3184-5p, hsa-miR-26a-2-3p, hsa-miR-218-2-3p, hsa-miR-150-5p, hsa-miR-125b-2-3p, and hsa-miR-125p. In concentric ellipses, the targets were recognized by 4, 5, 6, 7, 8 and 9 miRNAs (Fig. 2). The predicted results showed that FAM135A, IKZF2, PLAG1, ZNF148, PHC3 and LCOR were regulated by 8 miRNAs (Fig. 2, blue circles) and DYRK1A was regulated by 9 miRNAs (Fig. 2, purple circle).

**Figure 3:**
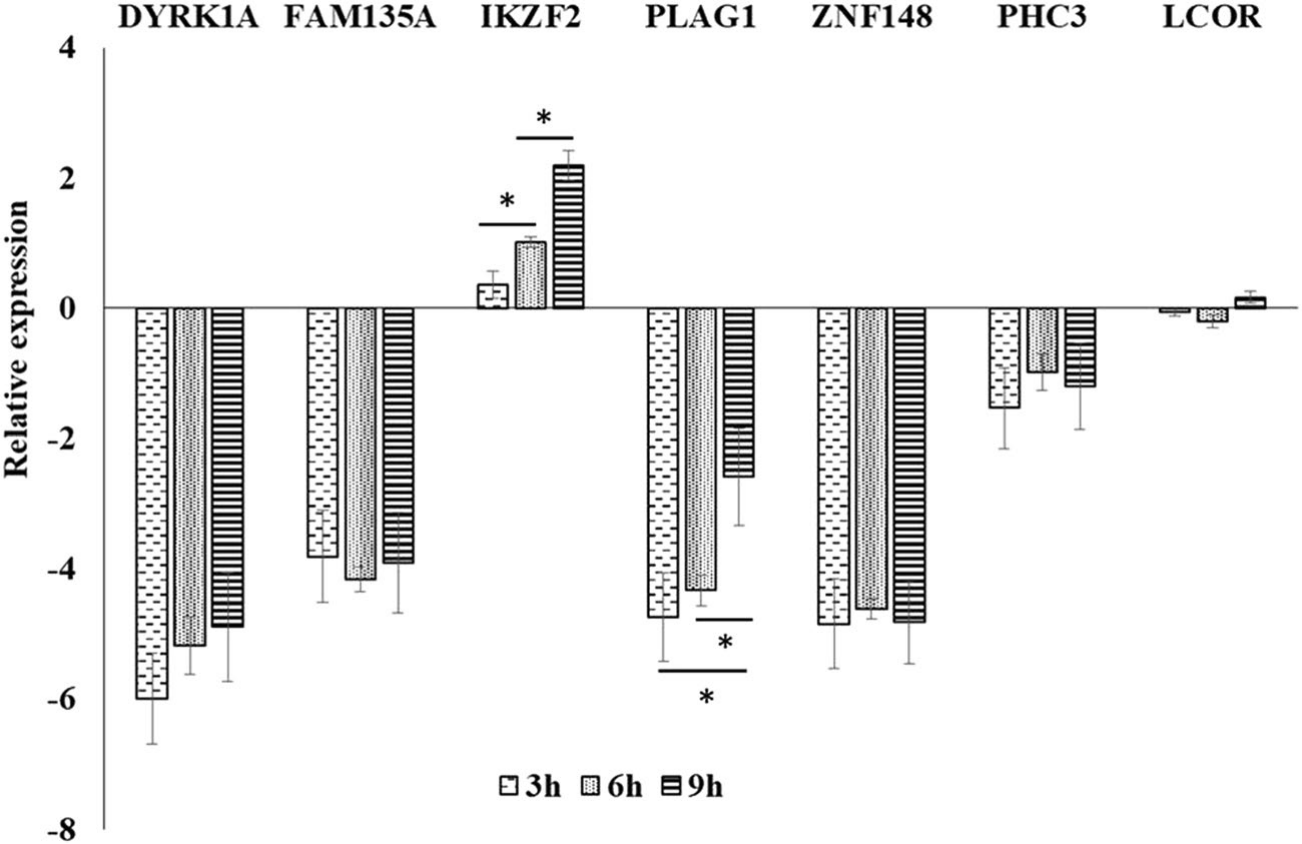
RT-qPCR validation of predicted target genes. DYRK1A, FAM135A, PLAG1, ZNF148, PHC3, LCOR and IKZF2 target genes were confirmed by RT-qPCR. Approximately 1.5 × 10^4^ cells were incubated for 12 h prior to infection with CVB3 (MOI = 1). After infected or uninfected cells of three repetitive wells were collected at 3 h, 6 h, and 9 h pi, isolation of total cellular RNA and RT-qPCR of each well was performed successively. Data normalization was performed using actin mRNA as an endogenous control. The relative amount of target mRNA was calculated by the log2 conversion of 2^−ΔΔCt^. Compared in two groups, significant differences were marked as *P < 0.05.

To verify the predicted results, the expression of the above 7 genes was detected by RT-qPCR from uninfected or CVB3-infected cells at 3 h, 6 h, and 9 h (pi). The results showed that *DYRK1A*, *FAM135A*, *PLAG1*, *ZNF148*, and *PHC3* were obviously inhibited at infection times of 3 h, 6 h, and 9 h pi (Fig. 3). There was no change in LCOR mRNA levels at each infection time point. However, the expression of *IKZF2*, which was recognized by 9 miRNAs (Fig. 2 purple circle), increased gradually with the prolongation of the infection time (Fig. 3).

## Discussion

Many studies have shown that miRNAs are key effector molecules during interactions between viruses and hosts^12,13,25^. To identify specific miRNAs involved in the cellular response of CVB3, miRNA profiles in CVB3-infected HeLa cells were generated.

In this study, a total of 597 differentially expressed miRNAs were identified across three time points in CVB3-infected HeLa cells; among them, both common and unique miRNAs were identified, indicating that expression of miRNA is complex during CVB3 virus infection. The 68 miRNAs and 34 novel miRNAs showed similar trends and indicated that these miRNAs simultaneously participated in interactions with host cells. The identified cellular miRNAs in infected cells can regulate many signal pathways to disturb viral replication from some reports, though we did not knock out these miRNAs to observe viral replication. Some of these miRNAs may be potential candidates for antiviral therapeutic or prophylactic targets.

Many reports show that viral infections can reshape the expression of cellular miRNAs, thereby affecting the microenvironment and metabolism of cells and ultimately leading to the pathogenesis of the virus. In this study, a total of 68 differentially expressed miRNAs were screened from RNA sequencing data. Then, we focused on functional studies from these known miRNAs. GO and KEGG analysis found that several biological processes were significantly related to cellular component, molecular function, biological process, etc., indicating that these differentially expressed miRNAs may be associated with CVB3 infection.

Then, 34 miRNAs were selected to construct miRNA-Target interactional networks by mirDIP and Navigator. The results showed that 7 target genes, including *FAM135A*, *IKZF2*, *PLAG1*, *ZNF148*, *PHC3*, *LCOR* and *DYRK1A*, were regulated by 8 miRNAs or 9 miRNAs. These genes are closely related to the regulation of cellular proliferation, differentiation, development and cellular growth control. The above results showed that CVB3 infection induced cellular miRNAs to regulate multiple cellular processes, which are important modulators of cellular proliferation, differentiation and development^26–30^.

CVB3 infection induced downregulation of DYRK1A and FAM135A, which are associated with neural system development. DYRK1A, as a member of the dual-specificity tyrosine phosphorylation-regulated kinase (DYRK) family, mainly regulates a signaling pathway of cell proliferation and differentiation of neuronal progenitor cells^31^. DYRK1A also activates the nuclear factor of activated T-cells (NFAT) to regulate HIV-1 transcription through NFAT nucleus translocation^32^. NFAT activation is also crucial to CVB3-induced myocarditis susceptibility^33^. Some research still shows that DYRK kinases play an important regulatory role in Herpesvirus replication. DYRK1A can significantly inhibit the replication of many Herpes viruses, such as HCMV, HSV-1, VZV and RhCMV, through either knockdown of DYRK1A or DYRK inhibitors^34,35^. Thus, we speculate that downregulated DYRK1A cannot promote CVB3 replication in this study, though further experimentation needs to be confirmed.

Although previous studies evaluated the biological relevance of *Fam135a* in the effects of ethanol on anxiety^36^, Fam135a has been shown to be differentially expressed in the amygdala, hypothalamus, pituitary, hippocampus, ventral tegmental area (VTA), and adrenal gland pituitary gland in mice^37^ and indicated that *FAM135A* is associated with neural system development. However, there is no report that *FAM135A* directly affects viral replication or participates in CVB3-infected pathophysiology.

Thus, our results may explain why CVB3 infection induces many miRNAs to inhibit the expression of *DYRK1A* and *FAM135A*, prevent neurodevelopment, and reduce cognitive function or severe neurological complications. This may be one of the important reasons for pathological changes in the nervous system caused by enterovirus.

miRNAs also participate and stabilize myogenesis, hematopoiesis, and neural development through regulation of gene transcription^38^. Zinc finger protein 148 (ZNF148, or ZFP-148), a Krüppel-type zinc finger transcription factor, is universally expressed in nearly all mammalian cells^39^. Multifunctional ZNF148 is involved in the regulation of upstream signaling pathways of cellular proliferation, apoptosis and embryogenesis^40,41^. To date, more than 30 target genes for ZNF148 have been identified, many of which can be associated with the clinical phenotypes observed in patients^42^. ZNF-148 also binds with miRNA to regulate CVB3 replication and pathogenicity^19,43^. A study showed that CVB3 replication increased in HeLa cells after ZNF148 was inhibited by miR-203^19^. Similarly, CVB3 infection also induced the upregulation of miR-20b to inhibit the expression of ZNF148 in CVB3-infected BALB/c mouse heart tissues^43^. Our results further confirmed that CVB3 infection induced many miRNAs to repress the expression of the transcriptional factors ZNF148.

Pleomorphic adenoma gene 1 (PLAG1), a member of the PLAG family of zinc finger transcription factors, changes the target gene transcription rate to regulate the expression of target genes. Regulation of Insulin-like growth factor-2 (IGF2) transcription to effect mTORC1 activity^44^ is the most important. PLAG1 also adjusts the transcription of the signal molecule of the Wnt pathway, regulators of the cell cycle, and immunomodulatory factors^45,46^. PLAG1 still stabilizes hematopoietic stem and progenitor cells (HSPCs)^47^. Abnormal misexpression of both PLAG1 and PLAGl2 appeared in acute myeloid leukemia-induced amphotropic murine leukemia virus (MLV) strain 4070A^48^. In combination with the above reports, CVB3 infection may break cellular signal transduction through inhibition of PLAG1, a topic that is important in future research.

CVB3 infection also inhibited the expression of transcriptional regulators such as polyhomeotic homolog 3 (PHC 3), which is a ubiquitous member of the human Polycomb complex^49^. PHC3 binds with the transcription factor E2F6 as a Polycomb complex to silence promoters of target genes to prevent cell cycle progression. Several studies have shown that proteins of some viruses may subvert a critical cellular defense mechanism through E2F6. In addition to simian virus 40 T antigen and adenovirus E1A, HPV E7 proteins inhibit the formation of Polycomb complexes to inactivate the transcriptional repression activity of E2F6^50^, resulting in the prolonged S-phase in HPV-infected cells. EBNA3C binds with the amino and carboxy termini of E2F6 to competitively inhibit the formation of E2F6/Polycomb complexes. Then, E2F6 is taken by EBNA3C to the E2F1 promoter to promote cell proliferation^51^.

Ligand-dependent corepressor (LCoR), as a regulator of nuclear receptor transactivation, binds histone deacetylases and C-terminal binding proteins (CtBP) to repress target gene transcription^52–54^. However, until now, there has been no report on the effect of LCoR on viral replication and pathogenesis, though expression of LCoR showed no significant change by RT-qPCR in this study.

Thus, CVB3 infection may induce HeLa cellular miRNA to inhibit the above transcriptional factors and transcriptional regulators to inhibit cell proliferation and development, though further studies will be explored in animal models.

miRNAs play a posttranscriptional regulatory role through targeting the 3′-untranslated region (UTR) of mRNA to cause mRNA degradation or translation repression^10,11^. However, many studies have shown that miRNAs induce gene activation by binding long non-coding RNAs (lncRNAs) or promoter-associated RNAs (pRNAs) or the transcriptional activator complex^55^. Our results indicated that CVB3 infection may induce miRNA-guided transcriptional gene *IKZF2* activation. IKZF2 (known as Helios), a member of the Ikaros family of Krüppel-like zinc finger proteins, plays a key role in the differentiation and activation of T cell functions^56,57^. IKZF2 is also associated with the regulation of inflammatory and immune responses^58,59^. To a certain extent, the IKZF2 protein still stabilizes regulatory T-cells (Tregs) suppressive function in the face of inflammatory responses regulatory^60,61^. Tregs, which express highly suppressive IKZF2, may be conducive to control of HIV replication^62^. Thus, it is possible that CVB3 infection boosts Treg cell function to inhibit both TFH (Follicular helper T cell) and Th1 effector cell responses through *IKZF2*. These may also be an important mechanism of enterovirus pathogenesis and destruction of immune responses influenced by miRNAs, though further studies are needed.

In this study, a new method for screening target genes of microRNA regulation was established in viral-infected cells by small-RNA high-throughput sequencing. In contrast to the previous study, we used highly differentially expressed microRNAs to construct a microRNA-Target interactional network through known microRNA databases. Using this network, we found that many target genes were recognized simultaneously by multiple microRNAs, and then the expression of these genes was identified by RT-qPCR detection. Six genes were identified to have significantly differential expression using this method. Our results further confirm that the expression of ZNF148 was repressed by microRNA during CVB3 infection, as previously reported^19,43^. Downregulation of 4 miRNA target genes, including *PLAG1*, *ZNF148*, *PHC3*, and *DYRK1A*, was first found in CVB3-infected cells, though 4 genes were associated with nonenterovirus replication and infection. We also found that the expression of *FAM135A* was significantly inhibited in CVB3-infected HeLa cells.

In conclusion, miRNA expression profiles and specific interactional networks were constructed in CVB3-infected HeLa cells by small-RNA sequencing technology in this study. Seven target genes, including *FAM135A*, *IKZF2*, *PLAG1*, *ZNF148*, *PHC3*, *LCOR* and *DYRK1A*, were recognized by 8 miRNAs or 9 miRNAs through miRNA-Target interactional networks. The RT-qPCR results showed that the expression of *DYRK1A*, *FAM135A*, *PLAG1*, *ZNF148*, and *PHC3* was obviously inhibited at 3 h, 6 h, and 9 h pi. However, the expression of *LCOR* did not show a significant change, and the expression of *IKZF2* increased with the prolongation of infection time in this study. Though our results are fairly limited and require extensive exploration on cardiomyocytes and animal model, this study will provide some information for miRNA-mediated regulatory mechanisms during enterovirus infection.

## Methods

### Cell & virus

HeLa cells were propagated in Dulbecco’s modified Eagle’s medium (DMEM) containing 10% fetal bovine serum (FBS) (GIBCO, Thermofisher, USA), 100 u/ml penicillin, and 100 μg/ml streptomycin at 37 °C. CVB3 M strain (MOI = 1) was propagated in HeLa cells with 2% FBS at 37 °C as in a previous study^63,64^.

### RNA isolation

Total RNA from 4 samples of CVB3-infected HeLa cells at 3 h, 6 h and 9 h pi and uninfected HeLa cells was isolated using TRIzol reagent (Thermofisher, USA) according to the manufacturer’s instructions. The enrichment of the miRNA fraction was performed using the PureLink miRNA Isolation Kit (Invitrogen, USA). RNA samples were subjected to construct the sequencing library. After the library was constructed, the quality of the library was assessed using a Bioanalyzer 2100 before carrying out NGS. A substantial amount of sRNA sequences (16-30 nt) were obtained after removal of linkers and low-quality data. After performing quality control, 4 samples with an optimal quality profile were prepared for NGS.

### Next-generation sequencing

Small-RNA sequencing using BGISEQ-500 technology was performed by BGI Co., Ltd, China. To obtain highly reliable sequencing data, strict quality control in each experiment step was carried out. After obtaining raw data, trimming was performed to remove adaptor sequences. Then, the miRNAs were annotated using the miRBase platform (release 20.0, *Homo sapiens*). The expression level of miRNAs was calculated using TPM (Transcripts Per Kilobase Million)^65^. The 49 nt sequence tags from HiSeq sequencing were first generated using data cleaning analysis to obtain credible clean tags. The clean tags were annotated into known miRNA categories, and no annotated tags were used to predict the novel miRNA. The differentially expressed miRNA was calculated by the log2 conversion of TPM ratio between infected cells and control cells.Hierarchical clustering of differentially expressed sRNAs (DESs) was performed. After obtaining similar changes in miRNA at 3 h, 6 h and 9 h pi, the biological processes of GO enrichment and KEGG pathway were analyzed using WEGO software and KEGG database.

### Generation of predicted miRNA-transcript interaction networks

Thirty-four miRNAs induced by CVB3 infection were input into the *microRNA Data Integration Portal*^66,67^. Interaction networks were generated using target genes identified by miRDIP. The visualized networks between miRNAs and their predicted mRNA targets were constructed using *NAViGaTOR v2.14*^23,68,69^.

### Reverse transcription quantitative PCR (RT-qPCR)

The expression level of 16 randomly selected miRNAs was confirmed using miRNA-specific RT-qPCR assays. Briefly, after 4 samples were collected as described above, 500 ng of total cellular RNA from each sample was reverse transcribed using the TaqMan microRNA Reverse Transcription Kit (Applied Biosystems) according to the manufacturer’s instructions. qPCR was carried out on a 7500 FAST Real-Time PCR System. Each sample was repeated three times. Data normalization was performed using U6 mRNA as an endogenous control. The relative amount of target mRNA was calculated by the log2 conversion of 2^−ΔΔCt^.

Based on the predicted target genes recognized by 8-9 miRNAs, target genes, including *DYRK1A*, *FAM135A*, *PLAG1*, *ZNF148*, *PHC3*, *LCOR* and *IKZF2*, were confirmed by RT-qPCR using a real-time PCR system (CFX96, USA). After approximately 1.5 × 10^4^ cells were incubated in each well of a 96-well cell plate for 12 h, HeLa cells were inoculated with CVB3 (MOI = 1). After infected or uninfected cells of three repetitive wells were collected at 3 h, 6 h, and 9 h pi, total cellular RNA of each well was isolated with TRIzol reagent (Invitrogen™, USA) according to the manufacturer’s instructions. RT-qPCR was performed using the One Step SYBR®PrimeScript^TM^ PLUS RT-PCR Kit (TAKARA, Japan) according to the manufacturer’s recommendations. RT-qPCR cycling was performed at 95 °C for 10 s, followed by 30 s at 95 °C and 30 s at 60 °C for 40 cycles. All gene primers are listed in Table S1. Data normalization was performed using actin mRNA as an endogenous control. The relative amount of target mRNA was calculated by the log2 conversion of 2^−ΔΔCt^.

### Statistics

Significant variability among the mean values (±standard deviations (SD)) of the experimental groups was determined by paired Student’s t test using SPSS 10.0 software (SPSS, Inc., Chicago, IL, USA). The differences were considered statistically significant at P < 0.05.

## Supplementary information

Supplementary information
