# Supplementary material for: Construction of miRNA-target networks using microRNA profiles of CVB3-infected HeLa cells
Source: Sci Rep. 2019 Nov 29;9:17876. doi: 10.1038/s41598-019-54188-w (PMC6884461; doi:10.1038/s41598-019-54188-w)
Supplement: Supplementary file 1 — Supplementary information [file 41598_2019_54188_MOESM1_ESM.pdf]

Supplementary Files: Construction of miRNA-target networks using microRNA profiles of CVB3-infected HeLa cells

Hai L-Yao[1], Mi Liu[1,2,+], Wen J-Wang[2], Xin L-Wang [2], Juan Song[2], Qin Q-Song[2], Jun Han[2,*]

Supplement

Table S1. Target gene primers of co-express regulatory networks from microRNA profiles induced by CVB3 infection

No	Name	Sequence	
1	DYRK1A	Forward	CAGGTGCGTCAGCAATTTCC
		Reverse	ATGCATTCTGTTGAGGGGCT
2	FAM135A	Forward	AAGTTCCTCTGCTGGCATCC
		Reverse	CCCCAGGCAATCCAAGTTCA
3	IKZF2	Forward	TGGCCCCATCTCTCTCATCA
		Reverse	ACTGGTGGTCATCATGGCTG
4	PLAG1	Forward	AAGCCAAGTCGTAAGAGCGT
		Reverse	TGCCTTCTGTTCTCTTGCGT
5	ZNF148	Forward	CAAAGTCTTGGGCATCGTGG
		Reverse	CTCACGTTCTCTTTCCCGCT
6	PHC3	Forward	TGGAGTTACAAGGTGGCGTG
		Reverse	TAGGGAGAGGTCTCCTGCAC
7	LCOR	Forward	CAAGCTCCCAAGCATCCTCA
		Reverse	GGTGGCATAGACCTGCTTGT

Table S2. Differential expression of miRNAs after CVB3 infection at 3h, 6h and 9h

No	Name	3h	P-value	6h	P-value	9h	P-value
1	hsa-miR-378i	15.58167	0	15.88035	0	15.86713	0
2	hsa-miR-518e-3p	12.60502	1.29E-44	6.491853	0.24967	7.022368	0.1218014
3	hsa-miR-422a	10.57932	3.01E-11	5.643856	0.49956	8.022368	0.01493204
4	hsa-miR-548au-3p	10.53138	1.51E-11	10.31061	1.84E-09	7.409391	0.0605074
5	hsa-miR-20b-3p	9.531381	3.89E-06	9.891784	1.18E-07	5.321928	0.493562
6	hsa-miR-150-5p	9.179909	0.000123747	7.491853	0.0623628	5.321928	0.493562
7	hsa-miR-4684-3p	9.076816	0.000247254	8.076816	0.015577	9.228819	5.54E-05
8	hsa-miR-6775-5p	8.366322	0.00394066	7.84549	0.0311676	7.022368	0.1218014
9	hsa-miR-4451	8.179909	0.01573196	5.643856	0.49956	5.321928	0.493562
10	hsa-miR-548i	7.321928	0.0628052	8.076816	0.00024275	7.022368	0.1218014
11	hsa-miR-299-3p	7.321928	0.0628052	7.129283	0.1247804	6.491853	0.245186
12	hsa-miR-765	6.906891	0.1254882	7.129283	0.1247804	8.022368	0.01493204
13	hsa-miR-3940-5p	5.826548	8.49E-16	4.768184	2.14E-07	4.629357	1.33E-06
14	hsa-miR-374b-5p	5.649061	0	3.257956	3.25E-203	3.209923	1.17E-193
15	hsa-miR-6873-5p	5.321928	0.500976	7.129283	0.1247804	7.409391	0.0605074
16	hsa-miR-26a-2-3p	4.852262	0	4.469755	0	4.669873	0
17	hsa-miR-99a-5p	4.585602	0	4.805087	0	1.803751	0
18	hsa-miR-196b-5p	4.447331	0	4.248992	0	5.468868	0
19	hsa-miR-16-2-3p	4.331957	0	4.99883	0	5.231417	0
20	hsa-miR-3613-5p	4.324867	0	4.295793	0	5.070016	0
21	hsa-let-7g-5p	4.240458	0	4.644235	0	4.885215	0
22	hsa-miR-186-3p	4.203872	0	4.840174	0	5.33039	0
23	hsa-miR-4284	4.044394	1.35E-07	5.224002	2.85E-19	3.965784	3.88E-07
24	hsa-miR-1250-5p	3.830223	1.12E-54	4.034003	4.00E-64	4.199156	1.32E-74
25	hsa-miR-5701	3.826548	4.51E-13	1.754888	0.0074969	1.70044	0.01987672
26	hsa-miR-4485-5p	3.815917	8.99E-10	2.77259	0.000849334	3.156504	2.38E-05
27	hsa-miR-181d-3p	3.70548	0	4.082628	0	4.884288	0
28	hsa-miR-195-5p	3.396129	1.90E-19	6.870365	5.51E-279	5.759333	1.58E-125
29	hsa-miR-4512	3.369234	7.87E-07	1.77259	0.0571586	1.969626	0.0330584
30	hsa-miR-548j-5p	3.217231	8.56E-10	3.689299	3.82E-14	3.270529	3.39E-10
31	hsa-miR-218-2-3p	3.217231	4.38E-09	3.812498	1.78E-15	4.495056	1.99E-25
32	hsa-miR-4634	3.209453	0.0118239	3.97728	0.00051559	2.087463	0.212752
33	hsa-miR-376a-3p	3.044394	1.55E-05	3.426265	1.89E-06	2.285402	0.001830568
34	hsa-miR-96-3p	2.85849	0	4.118741	0	2.813973	0
35	hsa-miR-4419b	2.624491	0.000869148	2.297681	0.0074969	1.5025	0.1398512
36	hsa-miR-25-5p	2.506483	8.58E-198	2.388179	2.30E-168	3.305016	0
37	hsa-miR-4747-3p	2.392317	0.125641	2.754888	0.038939	4.629357	1.33E-06
38	hsa-miR-4743-3p	2.380822	0.1806492	2	0.1090664	1.643856	0.442596
39	hsa-miR-188-3p	2.320544	1.02E-60	3.910806	0	2.530315	2.85E-78

40	hsa-miR-378d	2.266787	1.73E-15	2.639039	8.41E-23	2.040746	3.32E-12
41	hsa-miR-4763-3p	2.044394	0.0659024	2.169925	0.0384328	2.906891	0.0021564
42	hsa-miR-551b-3p	2	0.219666	1.807355	0.37445	1.70044	0.366978
43	hsa-miR-4734	2	0.219666	2.169925	0.218338	3.087463	0.0203742
44	hsa-miR-663a	1.928917	4.69E-07	2.454378	1.65E-11	2.589579	3.85E-13
45	hsa-miR-1268b	1.920615	0	3.875051	0	2.168067	0
46	hsa-miR-92b-5p	1.80762	9.07E-234	2.527705	0	1.972049	1.36E-288
47	hsa-miR-125b-2-3p	1.687209	0	2.933581	0	2.103176	0
48	hsa-miR-4721	1.643856	0.1806492	2.754888	0.00734872	2.554589	0.01210302
49	hsa-miR-664b-5p	1.584963	0.37622	1.807355	0.218338	3.70044	0.001692538
50	hsa-miR-105-5p	1.470795	1.50E-139	1.851034	5.31E-247	2.497691	0
51	hsa-miR-99b-5p	1.456317	0	4.205854	0	2.197358	0
52	hsa-miR-6804-5p	-1.53434	0.01890076	-1.86876	0.00410524	-2	0.00450544
53	hsa-miR-4726-5p	-1.69515	0.0263024	-3.76553	0.000276488	-2.38702	0.00482224
54	hsa-miR-105-3p	-1.83423	1.84E-250	-1.54315	5.61E-194	-1.82382	2.52E-244
55	hsa-miR-548az-5p	-2.06935	2.14E-08	-1.94382	2.30E-08	-2.55915	1.50E-10
56	hsa-miR-449c-5p	-2.32193	0.217838	-7.64386	0.0626374	-2.32193	0.224864
57	hsa-miR-374b-3p	-2.37762	0	-5.27512	0	-4.94097	0
58	hsa-miR-548b-3p	-2.42321	2.03E-07	-5.14568	5.21E-12	-2.56071	2.65E-07
59	hsa-miR-125b-1-3p	-2.59999	0	-2.41896	0	-1.84312	0
60	hsa-miR-4694-3p	-2.66297	0.00146753	-2.44057	0.01068564	-4.24793	8.52E-05
61	hsa-miR-218-5p	-2.69515	2.36E-06	-1.72991	0.000172081	-2.18057	3.02E-05
62	hsa-miR-3184-5p	-2.70774	0	-3.21293	0	-2.92983	0
63	hsa-miR-3622a-5p	-3.62449	6.34E-95	-3.08392	3.55E-82	-5.70541	1.68E-124
64	hsa-miR-151b	-3.64053	1.84E-291	-4.82052	0	-3.46773	7.27E-277
65	hsa-miR-188-5p	-4.23759	3.62E-137	-1.74482	6.96E-54	-2.57897	3.31E-86
66	hsa-miR-32-5p	-4.41971	0	-3.93542	0	-1.58459	2.02E-204
67	hsa-miR-26a-1-3p	-6.23096	0	-3.2416	0	-5.56417	0
68	hsa-miR-522-5p	-12.6751	9.39E-48	-12.6751	1.17E-47	-7.35315	2.38E-45

Table S3. Differential expression of 16 miRNAs were confirmed by RT-qPCR after CVB3 infection at 3h, 6h and 9h

No	miRNA	Methods	3h	6h	9h
1	hsa-miR-99b-5p	RT-PCR	1.36	2.02	2.58
		small-RNA sequence	4.205854	2.197358	1.456317
2	hsa-miR-99a-5p	RT-PCR	1.38	1.45	2.52
		small-RNA sequence	4.585602	4.805087	1.80375
3	hsa-let-7f-1-3p	RT-PCR	1.37	1.42	1.83
		small-RNA sequence	3.851848	1.713054	4.41349
4	hsa-miR-92b-5p	RT-PCR	1.42	1.59	1.97
		small-RNA sequence	1.80762	2.527705	1.97205
5	hsa-miR-378i	RT-PCR	1.60	1.29	4.11
		small-RNA sequence	15.58167	15.88035	15.8671
6	hsa-miR-374b-5p	RT-PCR	1.20	1.54	2.00
		small-RNA sequence	5.649061	3.257956	3.20992
7	hsa-miR-26a-2-3p	RT-PCR	2.61	1.35	1.19
		small-RNA sequence	4.852262	4.469755	4.66987
8	hsa-miR-26a-1-3p	RT-PCR	-1.54	-1.55	-1.40
		small-RNA sequence	-6.23096	-3.2416	-5.5642
9	hsa-miR-25-5p	RT-PCR	2.95	1.33	3.12
		small-RNA sequence	2.506483	2.388179	3.30502
10	hsa-miR-196b-5p	RT-PCR	1.32	1.25	1.73
		small-RNA sequence	4.447331	4.248992	5.46887
11	hsa-miR-186-3p	RT-PCR	2.24	2.69	3.43
		small-RNA sequence	4.203872	4.840174	5.33039
12	hsa-miR-16-2-3p	RT-PCR	1.39	1.42	2.32
		small-RNA sequence	4.331957	4.99883	5.23142
13	hsa-miR-1296-5p	RT-PCR	1.49	1.78	2.09
		small-RNA sequence	2.760157	3.471105	1.58859
14	hsa-miR-125b-2-3p	RT-PCR	1.86	1.05	1.71
		small-RNA sequence	1.687209	2.933581	2.10318
15	hsa-miR-1268bp	RT-PCR	1.28	2.78	2.04
		small-RNA sequence	1.920615	3.875051	2.168067
16	hsa-let-7g-5p	RT-PCR	1.53	1.45	1.74
		small-RNA sequence	4.240458	4.644235	4.88521

Table S4. GO categories were identified in CVB3 infected HeLa cells

Ontoloty	GO term	Number of Genes
biological_process	biological adhesion	322
biological_process	biological regulation	3119
biological_process	cell killing	22
biological_process	cellular component organization or biogenesis	1382
biological_process	cellular process	5148
biological_process	developmental process	1857
biological_process	establishment of localization	1550
biological_process	growth	279
biological_process	hormone secretion	70
biological_process	immune system process	483
biological_process	localization	1837
biological_process	locomotion	358
biological_process	metabolic process	4184
biological_process	multi-organism process	510
biological_process	multicellular organismal process	2171
biological_process	negative regulation of biological process	540
biological_process	positive regulation of biological process	708
biological_process	regulation of biological process	2911
biological_process	reproduction	403
biological_process	reproductive process	387
biological_process	response to stimulus	2332
biological_process	rhythmic process	47
biological_process	signaling	1554
biological_process	single-organism process	4508
cellular_component	cell	5751
cellular_component	cell junction	190
cellular_component	cell part	5751
cellular_component	collagen trimer	15
cellular_component	extracellular matrix	162
cellular_component	extracellular matrix part	27
cellular_component	extracellular region	322
cellular_component	extracellular region part	312
cellular_component	macromolecular complex	1270
cellular_component	membrane	3059
cellular_component	membrane part	2493
cellular_component	membrane-enclosed lumen	768
cellular_component	organelle	4422
cellular_component	organelle part	1998
cellular_component	synapse	60
cellular_component	synapse part	35

cellular_component	virion	2
cellular_component	virion part	1
molecular_function	antioxidant activity	24
molecular_function	binding	5846
molecular_function	catalytic activity	2532
molecular_function	channel regulator activity	37
molecular_function	electron carrier activity	2
molecular_function	enzyme regulator activity	406
molecular_function	guanyl-nucleotide exchange factor activity	68
molecular_function	metallochaperone activity	1
molecular_function	molecular transducer activity	766
molecular_function	nucleic acid binding transcription factor activity	493
molecular_function	protein binding transcription factor activity	197
molecular_function	receptor activity	380
molecular_function	receptor regulator activity	6
molecular_function	structural molecule activity	158
molecular_function	translation regulator activity	11
molecular_function	transporter activity	511

Table S4. KEGG pathways were identified in CVB3 infected HeLa cells

Pathway_level1	Pathway_level2	Number_of_Genes
Cellular Processes	Cell growth and death	304
Cellular Processes	Cell motility	271
Cellular Processes	Cellular community	626
Cellular Processes	Transport and catabolism	471
Environmental Information Processing	Membrane transport	41
Environmental Information Processing	Signal transduction	1494
Environmental Information Processing	Signaling molecules and interaction	517
Genetic Information Processing	Folding, sorting and degradation	364
Genetic Information Processing	Replication and repair	105
Genetic Information Processing	Transcription	148
Genetic Information Processing	Translation	190
Human Diseases	Antineoplastic resistance	139
Human Diseases	Cancers: Overview	1389
Human Diseases	Cancers: Specific types	327
Human Diseases	Cardiovascular diseases	173
Human Diseases	Endocrine and metabolic diseases	303
Human Diseases	Immune diseases	174
Human Diseases	Infectious diseases: Bacterial	523
Human Diseases	Infectious diseases: Parasitic	265
Human Diseases	Infectious diseases: Viral	481
Human Diseases	Neurodegenerative diseases	287
Human Diseases	Substance dependence	169
Metabolism	Amino acid metabolism	231
Metabolism	Biosynthesis of other secondary metabolites	6
Metabolism	Carbohydrate metabolism	224
Metabolism	Energy metabolism	37
Metabolism	Global and overview maps	1231
Metabolism	Glycan biosynthesis and metabolism	170
Metabolism	Lipid metabolism	195
Metabolism	Metabolism of cofactors and vitamins	210
Metabolism	Metabolism of other amino acids	92
Metabolism	Metabolism of terpenoids and polyketides	9
Metabolism	Nucleotide metabolism	664
Metabolism	Xenobiotics biodegradation and metabolism	41
Organismal Systems	Aging	133

Organismal Systems	Circulatory system	395
Organismal Systems	Development	337
Organismal Systems	Digestive system	425
Organismal Systems	Endocrine system	697
Organismal Systems	Environmental adaptation	116
Organismal Systems	Excretory system	254
Organismal Systems	Immune system	743
Organismal Systems	Nervous system	368
Organismal Systems	Sensory system	205

Table S5. 34 known differentially expressed miRNAs were selected to construct interaction networks

No.	miRNA	3h	6h	9h
1	hsa-miR-378i	15.58167	15.88035	15.86713
2	hsa-miR-548au-3p	10.53138	10.31061	7.409391
3	hsa-miR-150-5p	9.179909	7.491853	5.321928
4	hsa-miR-4684-3p	9.076816	8.076816	9.228819
5	hsa-miR-548i	7.321928	9.076816	7.022368
6	hsa-miR-3940-5p	5.826548	4.768184	4.629357
7	hsa-miR-374b-5p	5.649061	3.257956	3.209923
8	hsa-miR-26a-2-3p	4.852262	4.469755	4.669873
9	hsa-miR-196b-5p	4.447331	4.248992	5.468868
10	hsa-miR-16-2-3p	4.331957	4.99883	5.231417
11	hsa-miR-3613-5p	4.324867	4.295793	5.070016
12	hsa-let-7g-5p	4.240458	4.644235	4.885215
13	hsa-miR-186-3p	4.203872	4.840174	5.33039
14	hsa-miR-4284	4.044394	5.224002	3.965784
15	hsa-miR-1250-5p	3.830223	4.034003	4.199156
16	hsa-miR-5701	3.826548	1.754888	1.70044
17	hsa-miR-181d-3p	3.70548	4.082628	4.884288
18	hsa-miR-195-5p	3.396129	6.870365	5.759333
19	hsa-miR-548j-5p	3.217231	3.689299	3.270529
20	hsa-miR-218-2-3p	3.217231	3.812498	4.495056
21	hsa-miR-25-5p	2.506483	2.388179	3.305016
22	hsa-miR-4747-3p	2.392317	2.754888	4.629357
23	hsa-miR-378d	2.266787	2.639039	2.040746
24	hsa-miR-663a	1.928917	2.454378	2.589579
25	hsa-miR-92b-5p	1.80762	2.527705	1.972049
26	hsa-miR-125b-2-3p	1.687209	2.933581	2.103176
27	hsa-miR-548az-5p	-2.06935	-1.94382	-2.55915
28	hsa-miR-374b-3p	-2.37762	-5.27512	-4.94097
29	hsa-miR-125b-1-3p	-2.59999	-2.41896	-1.84312
30	hsa-miR-4694-3p	-2.66297	-2.44057	-4.24793
31	hsa-miR-3184-5p	-2.70774	-3.21293	-2.92983
32	hsa-miR-3622a-5p	-3.62449	-3.08392	-5.70541

33	hsa-miR-151b	-3.64053	-4.82052	-3.46773
34	hsa-miR-32-5p	-4.41971	-3.93542	-1.58459
